# Optimization of 3D Printing Parameters for Enhanced Surface Quality and Wear Resistance

**DOI:** 10.3390/polym15163419

**Published:** 2023-08-16

**Authors:** Alexandra Ileana Portoacă, Razvan George Ripeanu, Alin Diniță, Maria Tănase

**Affiliations:** Mechanical Engineering Department, Petroleum-Gas University of Ploiești, 100680 Ploiești, Romania; alexandra.portoaca@upg-ploiesti.ro (A.I.P.); rrapeanu@upg-ploiesti.ro (R.G.R.)

**Keywords:** ABS, PLA, 3D printing, friction coefficient, roughness, wear, infill percentage, layer thickness, parametric optimization, design of experiments

## Abstract

In recent years, there has been a growing interest in the field of 3D printing technology. Among the various technologies available, fused deposition modeling (FDM) has emerged as the most popular and widely used method. However, achieving optimal results with FDM presents a significant challenge due to the selection of appropriate process parameters. Therefore, the objective of this research was to investigate the impact of process parameters on the tribological and frictional behavior of acrylonitrile butadiene styrene (ABS) and polylactic acid (PLA) 3D-printed parts. The design of experiments (DOE) technique was used considering the input design parameters (infill percentage and layer thickness) as variables. The friction coefficient values and the wear were determined by experimental testing of the polymers on a universal tribometer employing plane friction coupling. Multi-response optimization methodology and analysis of variance (ANOVA) were used to highlight the dependency between the coefficient of friction, surface roughness parameters, and wear on the process parameters. The optimization analysis revealed that the optimal 3D printing input parameters for achieving the minimum coefficient of friction and linear wear were found to be an infill percentage of 50% and layer thickness of 0.1 mm (for ABS material), and an infill percentage of 50%, layer thickness of 0.15 mm (for PLA material). The suggested optimization methodology (which involves minimizing the coefficient of friction and cumulative linear wear) through the optimized parameter obtained provides the opportunity to select the most favorable design conditions contributing to a more sustainable approach to manufacturing by reducing overall material consumption.

## 1. Introduction

Fused deposition modeling (FDM) stands out as the most prevalent additive manufacturing method, operating by constructing objects layer by layer. This process involves the incremental deposition of filaments to create the desired object, and it has the capacity to handle a broad spectrum of materials, including polylactic acid (PLA), polycarbonate, poly-caprolactone, acrylonitrile butadiene styrene, and composite materials with polymers [1,2,3].

Extensive research has been conducted in the field of 3D printing to investigate the mechanical properties of parts fabricated using these technologies. Multiple studies [4,5,6,7,8,9,10,11,12,13,14,15,16] have explored how factors such as layer thickness, layer height, infill density, and other parameters affect the mechanical properties of these parts. Other studies [6,17,18,19,20,21,22,23,24,25,26] are investigating the influence of printing parameters on the dimensional accuracy of 3D-printed parts. Additionally, post-processing heat treatments can be used to further enhance the quality and performance of the printed parts [27,28,29,30,31,32,33,34,35,36,37,38,39]. These conditions significantly influence the properties exhibited by the printed components, and therefore many experimental tests should be carried out in order to obtain the optimal parameters for each analyzed physical or mechanical property. Optimization techniques play a crucial role in identifying optimal parameters for enhanced performance. Subsequently, researchers [40,41,42,43,44,45,46,47,48,49] have applied various optimization techniques to refine the parameters directly affecting the mechanical performance of 3D-printed parts. Response surface methodology is a common method used in scientific papers [41,43,48,50,51,52,53,54,55] for optimization analysis. Grey relational analysis (GRA) is a multi-response optimization technique that draws on the principles of the Taguchi technique. Studies [44,56,57,58,59,60,61,62] have been conducted recently using GRA to enhance various responses by optimizing processing parameters for 3D printing materials.

The study proposed by [41] focuses on optimizing the printing process using the fused deposition modeling (FDM) method, considering infill percentage (IP), extruder temperature (ET), and layer thickness (LT) as variables, which are adjusted based on the design of experiments (DOE) principles. A total of 20 experiments are designed within the parameter ranges of 15–55% for IP, 190–250 °C for ET, and 0.15–0.35 mm for LT. The main output responses evaluated are the maximum failure load, weight, fabrication time, and surface roughness of the printed samples. The statistical analysis reveals that increasing the infill percentage (IP) and setting the extruder temperature (ET) at 220 °C led to an increase in the failure load of the samples. Furthermore, the optimization process aims to reduce both the weight and fabrication time of the specimens while achieving a maximum failure load and minimizing surface roughness.

The research of [42] investigates the impact of three process variables, namely layer thickness, infill percentage, and print speed, on the hardness and strength of PLA fabricated specimens based on the Taguchi L9 orthogonal array. The study unveiled that both layer thickness and infill percentage play a vital role in determining the mechanical properties of FDM structures.

The scientific work of [45] aimed to optimize the settings of a 3D printer using ABS material, considering several performance characteristics such as flexural strength, tensile strength, average surface roughness, print time, and energy consumption. The study focuses on three measurable characteristics: layer thickness, printing speed, and infill density. To determine the significance of each performance parameter, the researchers employed analysis of variance (ANOVA). Their findings revealed that achieving the desired surface roughness and print time primarily depends on the layer thickness, while infill density significantly influences the mechanical characteristics of the printed object. Rodriguez et al. [47] conducted an optimization and statistical analysis to examine the impact of various 3D printing parameters (geometric pattern, infill percentage, printing direction, and layer height) on the ultimate tensile stress (UTS) and modulus of elasticity (E) for PLA, ABS, and Nylon + CF manufactured by 3D FDM printing, using two designs of experiments systematically to investigate these effects. The main contribution of this work lies in identifying the printing parameters that maximize the UTS and determining which parameters are not significant for the three materials taken under consideration. The initial design of experiments revealed that the material type and infill percentage (33%, 66%, and 100%) significantly influence the print outcome, while the geometric internal pattern (tridimensional, hexagonal, and linear) is considered irrelevant and excluded from subsequent analysis. In a following DOE, it was discovered that reducing the layer height from 0.18 mm to 0.14 mm and adjusting the printing direction from 0°/90° to +45°/−45° leads to an increase in ultimate tensile stress (UTS) for all three materials.

A similar study was performed in [48] with the aim of optimizing the printing parameters (layer thickness, printing speed, and nozzle temperature) for ABS polymer to improve surface quality and reduce printing time. 

Mani et al. [63] assessed, with the Taguchi design, the impact of printing parameterslayer thickness (0.15 mm, 0.25 mm, and 0.35 mm), nozzle temperature (210 °C, 215 °C, and 220 °C), and infill density (55%, 60%, and 65%)on the tensile strength, hardness, and surface roughness of PLA material. To achieve the highest tensile strength, the optimal parameters were a layer thickness of 0.35 mm, an infill density of 65%, and a nozzle temperature of 220 °C. In terms of hardness, the optimal parameters were a layer thickness of 0.25 mm, an infill density of 65%, and a nozzle temperature of 215 °C. For surface roughness, the optimal parameters were a layer thickness of 0.15 mm, an infill density of 55%, and a nozzle temperature of 210 °C. 

However, there is a limited number of references, specifically [64,65,66,67,68,69,70], that explore the tribological behavior of 3D-printed parts. Additionally, there is scarce literature available on experimental studies that investigate the influence of printing variables, such as infill percentage and layer thickness, on the tribological and frictional behavior of 3D-printed components.

In a related study [71], the primary focus was examining the friction characteristics of 3D-printed samples. The study observed that the transverse direction of the printed samples exhibited higher coefficient of friction values compared to the longitudinal direction, regardless of the applied loads and sliding speeds. Furthermore, the study compared the friction behavior of two 3D-printed materials: PLA and ABS. Consistently, PLA samples demonstrated lower coefficient of friction values than ABS samples, regardless of the printing direction, applied loads, and sliding speeds. The objective of the research paper [65] was to examine how the scaffolding angle and raster gap influence friction behavior, specifically the coefficient of friction and wear rate. Moreover, graphite flakes were introduced into the ABS matrix to potentially enhance the material properties. The study demonstrated that the scaffolding angle only had a significant impact on behavior for a positive printing gap, whereas it showed no significant effect for a negative gap. The optimal combination, resulting in the highest friction coefficient and acceptable specific wear rates, was achieved with a scaffolding angle of 90° and a negative gap. Incorporating graphite into the material composition increased the coefficient of friction, but it led to a reduction in wear properties.

The main goal of the study [72] was to evaluate the wear rate of PLA by identifying the optimal parameters for 3D printing, namely extrusion temperature, fill density, and nozzle speed. The research concluded that infill percentage had the most significant impact on the wear rate, followed by extrusion temperature and nozzle speed. The optimal set of process parameters determined was an infill percentage of 100%, an extrusion temperature of 220 °C, and a nozzle speed of 40 mm/s. Perepelkina et al. [69] demonstrated that modifying the settings of 3D printing had a notable influence on the strength, stiffness, surface quality, and, subsequently, the tribological properties of the printed parts. The study found that the white filament color exhibited the highest friction tendency, whereas test pieces printed at a 45° angle orientation with black filament color revealed the maximum wear depth. Additionally, it was observed that wear reduced when the parts were subjected to sliding under high loads [64].

Frunzaverde et al. [2] investigated the influence of filament color on the characteristics of FFF-printed PLA materials, specifically in relation to dimensional accuracy, tensile strength, and friction properties and concluded that the optimal characteristics in terms of dimensional accuracy, tensile strength, and sliding wear behavior were within the temperature range of between 210 °C and 220 °C for natural PLA, while slightly lower temperatures (200–210 °C) were found to be optimal for black PLA. Both types of PLA, when printed at temperatures exceeding the upper limit of the aforementioned ranges, exhibited lower values of the ultimate tensile strength (UTS) and friction coefficient. 

Given the anisotropic nature of 3D prisms, the analysis of the breaking behavior is very important. Both experimental and numerical studies have been carried out, which have revealed that the load-bearing capacity of these parts is radically influenced by the orientation of the printing. Printing the part in the direction of the stress maximizes its integrity, while a 90° orientation considerably decreases the maximum stress force supported by the part [73,74,75]. Fracture toughness studies have also been conducted, employing fracture mechanics principles for 3D-printed parts. These studies obtained fracture toughness values that reached 1.97 MPa·m^1/2^ [75].

The current investigation aims to establish the relationship between printing process parameters and the tribological and frictional behavior of 3D-printed components (made of ABS and PLA materials) using fused deposition modeling (FDM). To achieve this aim, a full factorial design of the experiment method is used for efficient experimentation by simultaneously exploring multiple factors and their combinations. The selection of optimal values of input parameters for minimal values of both coefficient of friction and cumulative linear wear is obtained using the Minitab response optimizer and grey relational analysis algorithm. Additionally, Pareto charts and ANOVA present the main effect plots to highlight the influence of input variables on the surface roughness, coefficient of friction, and cumulative linear wear for both analyzed polymers. 

The analyzed 3D-printed materials have many practical applications, such as non-circular gears used in different industrial fields (robotics, automotive industry, medical devices, and textile industry). In the case of non-circular gears, having a complex geometric shape, classic manufacturing is more difficult to apply, thus justifying the choice of 3D printing technology, correlated with other advantages such as low weight, low noise, and the possibility of working in aggressive environments. The tribological behavior of gears has a direct impact on the friction occurring between their meshing teeth. Higher friction levels can result in energy losses, reduced efficiency, and increased wear, while lower friction levels contribute to enhanced performance and minimized power losses.

The performed investigation intended to highlight the influence of printing parameters on the coefficient of friction and wear, aiming for their minimization, resulting inlow energy consumption; therefore, a higher efficiency of the equipment in which the parts from the tested materials isused. Moreover, lower wear means higher durability, longer service life, and lower material consumption, resulting in reduced CO_2_ emissions both during the production of semi-finished products and during materials processing.

On the other hand, analyzing the specialized literature presented above, no scientific work has made a complete correlation between the printing parameters and the tribological behavior of 3D-printed parts in order to optimize the printing parameters.

## 2. Materials and Methods

**Figure 1:**
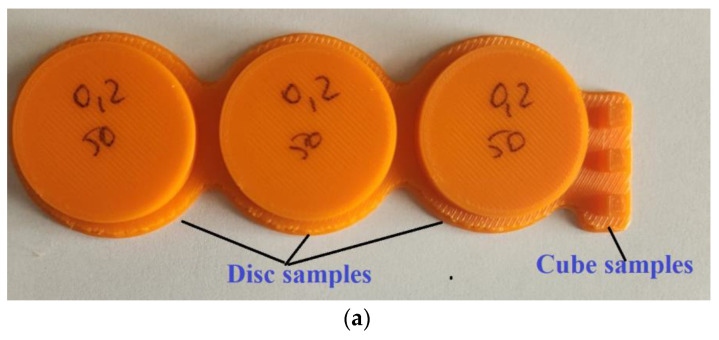

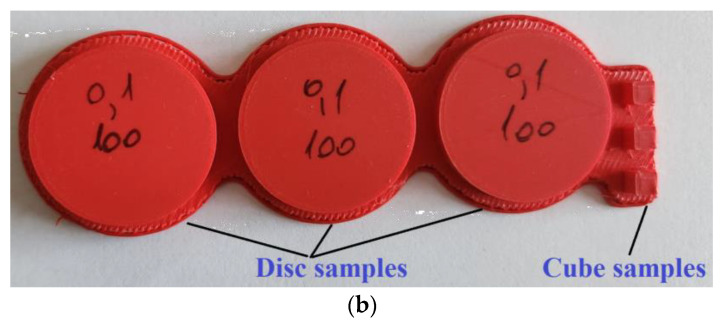
The shape of the tested samples: (**a**) ABS material and (**b**) PLA material.

For the experimental study, 108 samples were printed, comprising both flat disks and cubes. Disc and cube structures were considered to simulate contact on a flat surface similar to that specific to non-circular gears with curved teeth. The shape of the samples is depicted in Figure 1. ABS and PLA filaments were supplied by Polymaker (Utrecht, The Netherlands). The printing process involved various combinations of three different layer thicknesses (0.10 mm, 0.15 mm, and 0.20 mm) and three infill percentages (50%, 75%, and 100%). For each combination of printing parameters, three measurements were performed, and it was observed that the stabilization of the values for the coefficient of friction and linear wear was obtained at the same value as the measured data. Small differences (of the order of 10^−3^) of the friction coefficient values were recorded, these differences being insignificant considering the reporting mode of the friction coefficient values (with 2 digits).

**Figure 2:**
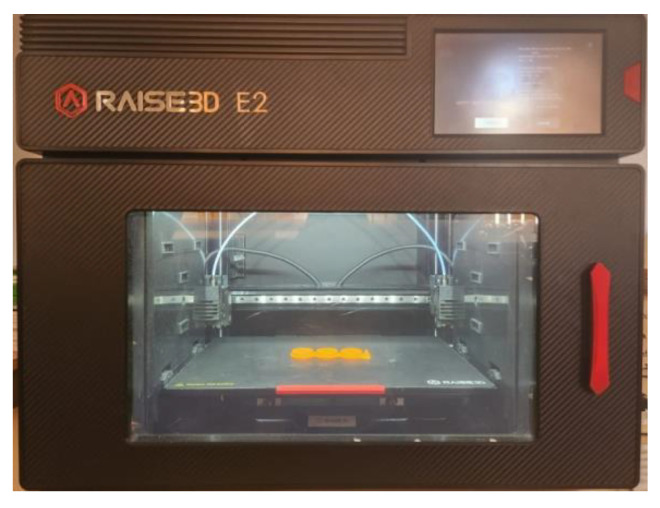
3D printed samples fabrication.

The Raise E2 3D printer (Irvine, CA, USA), having a volume capacity of 330 × 240 × 240 mm (Figure 2), was used for the printing process. The specific printing parameters for the present study (Table 1) were: build orientation X-Y model lines and 45° orientation.

In this study, the full factorial design method was used through Minitab 19 software to optimize the tribological behavior of ABS and PLA 3D-printed parts. The investigation focused on two input parameters, namely infill percentage and layer thickness, with three levels, as presented in Table 2.

The total number of experiments required is determined by the function of the number of input factors (n) and the number of levels (k). In this specific scenario, an orthogonal array consisting of 3^2^ tests was considered.

**Figure 3:**
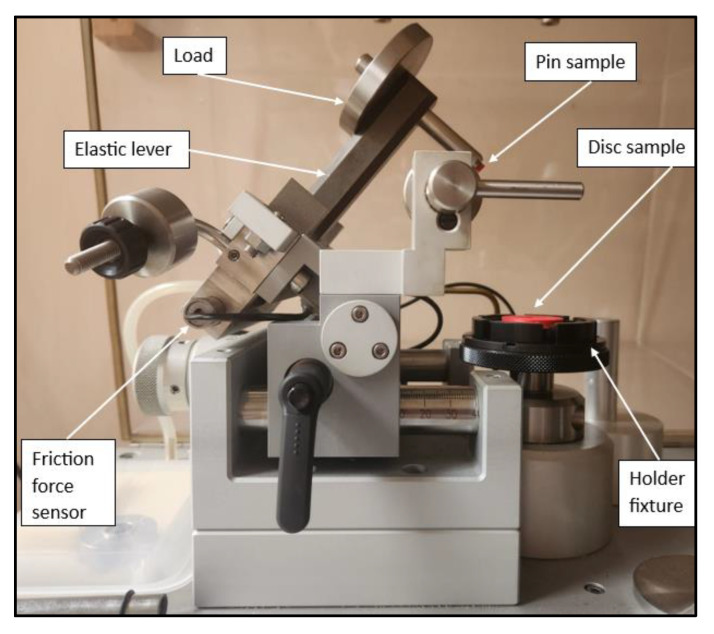
The test machine used to determine the sliding coefficient of friction.

The coefficients of friction were determined using a CSM Instruments THT (Freiburg im Breisgau, Germany) pin-on-disc tribometer, with the functional components described in Figure 3.The friction pair consisted of the disc sample (15 mm radius) and a cubic sample (4 mm side) of the same material (ABS and PLA), as presented in Figure 1. 

During the tribological test, the following parameters were used: normal load—7 N, friction length—50 m, and linear speed—0.314 m/s. The tests were performed at room temperature (20 °C) in air with 54% relative humidity. The coefficient of friction (μ) was calculated from the ratio of the tangential friction force and the normal force. Three friction pairs were tested for each combination of printing parameters. Continuous measurements were taken during the test to determine the coefficient of friction and cumulative linear wear (the linear wear of both disc and cube samples).

**Figure 4:**
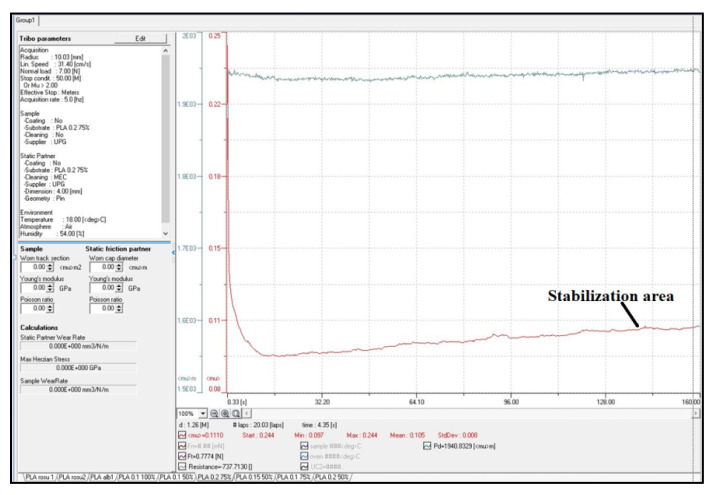
Measurement of friction coefficient and wear using InstrumX software.

The chosen friction coupling (contact on a flat surface) simulates the type of contact specific to non-circular gears with curved teeth. The friction length of 50 mm, considered in the experiments (based on the authors’ experience in the works [76,77]), corresponds to the value at which a stabilization of friction coefficient was obtained (as seen in Figure 4) and no influence of the temperature appears (the heat developed by friction phenomenon involves plastic materials change their mechanical characteristics by increasing the temperature). The sliding speed of 0.314 m/s corresponds to the sliding speed obtained on the AMSLER type A135 tribometer at a roller diameter of 30 mm and is close to the speed in non-circular gears from some practical applications. The load of 7N corresponds to a pressure of 0.435 MPa, representing approximately 4.6% (for ABS) and 2.52% (for PLA) of yield strength, respectively, compared to values established by the authors in the previous works [9,11].

**Figure 5:**
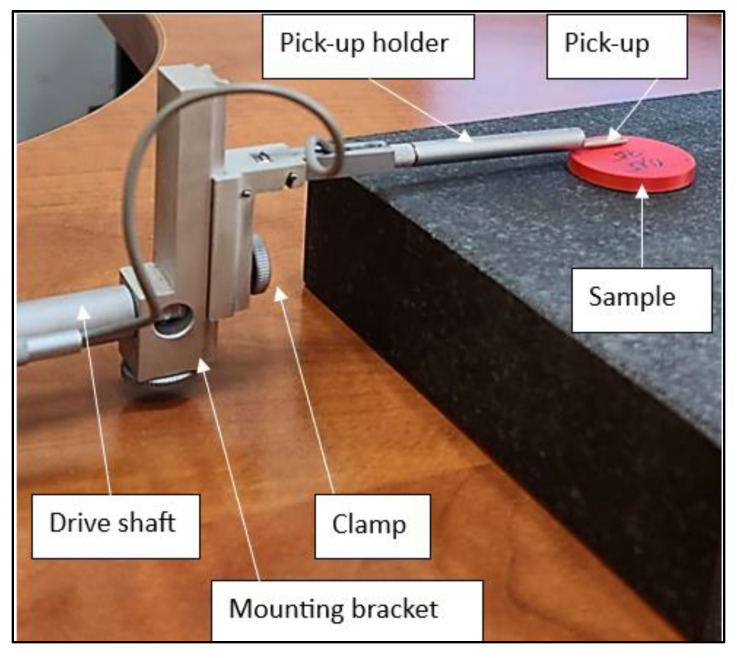
The surface roughness tester.

The surface roughness of the 3D-printed samples was assessed by measuring *R**_a_*** (arithmetic mean deviation), *R_t_* (total height of profile), and *R**_z_*** (average peak to valley height) values. This measurement was conducted using a Surtronic 3+ surface roughness tester (Taylor Hobson, Leichester, UK)with the functional components described in Figure 5.

**Figure 6:**
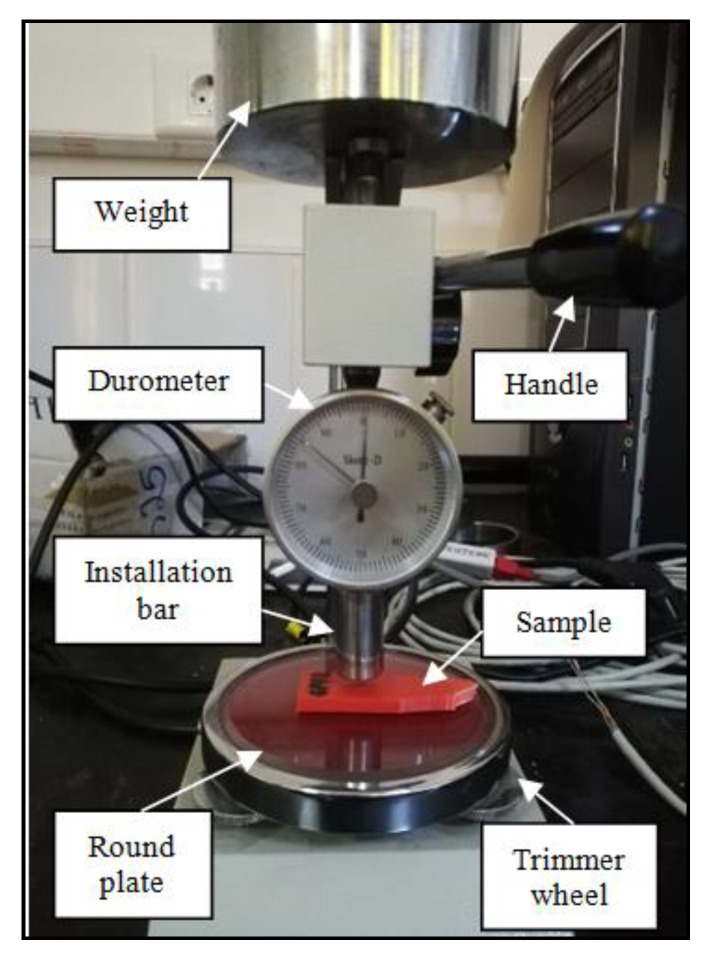
Shore D hardness testing device.

In addition, hardness testing was performed using a Shore D hardness tester (Wenzhou Tripod Instrument Manufacturing Co., Ltd., 15 Changsheng Road, Wenzhou, China) with a cone indentor30° Type D durometer, a specific method of harness measurement for polymers, according to standard ISO 7619-1:2010Rubber, vulcanized or thermoplastic—determination of indentation hardness—Part 1: Durometer method (Shore hardness). After applying sufficient pressure between the gauge and the material and ensuring the needle has reached its maximum depth of penetration, the hardness measurement was accurately displayed by the measurement needle (Figure 6).

**Figure 7:**
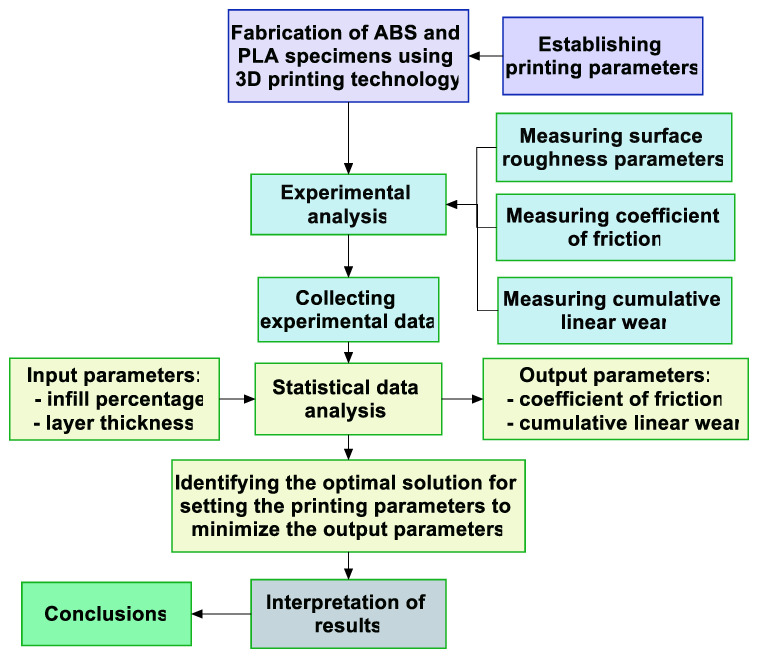
Flow chart for the performed analysis.

To provide a visual representation of the performed investigation, the flow chart from Figure 7 was created using the specialized software Clickcharts. 

Grey relational analysis was implemented to determine the optimal combination of independent variables that results in the lowest values both for the coefficient of friction and cumulative linear wear. Therefore, the smaller-is-better option should be considered and accordingly, the data were normalized using the following formula [57]:

(1) $$x_{ij}=\frac{\max(y_{ij})-y_{ij}}{\max(y_{ij})-\min(y_{ij})},$$

where *y_ij_* are the data points, and *x_ij_* are the resulting normalized data. 

The normalized data points were transformed to a deviation sequence Δ0i(k) by scaling them between 0 and 1, applying the equation from [57]:

(2) Δ0i(k)=x0(k)−Δxi(k),

where x0(k) represents the reference value and xi(k) represents the set of normalized data points. In this case, the reference value was fixed at 1. 

The grey relational coefficient $\varepsilon_{i}(k)$ is calculated as:

(3) $$\varepsilon_{i}(k)=\frac{\Delta \min+(\psi \cdot \Delta \max)}{\Delta ij+(\psi \cdot \Delta \max)}.$$

In Formula (3), Δmin and Δmax represent the minimum, respectively, the maximum values obtained for the deviation sequence responses. Each data point in the deviation sequence is denoted as Δ*ij*. In this particular study, a distinguishing coefficient ψ of 0.5 was used. The minimum deviation, Δmin, has a value of 0, while the maximum deviation, Δmax, has a value of 1.

For each experiment, the grey relational grade ***γ****_i_* is computed as a function of the grey relational coefficients $\varepsilon_{i}(k)$ and the number of response variables *n*, with the formula:

(4) $$\gamma_{i}=\frac{\sum_{i=1}^{n}\varepsilon_{i}(k)}{n}.$$

## 3. Results and Discussion

### 3.1. Experimental Determination of Roughness Parameters, Coefficient of Friction, Cumulative Linear Wear, and Hardness

**Figure 8:**
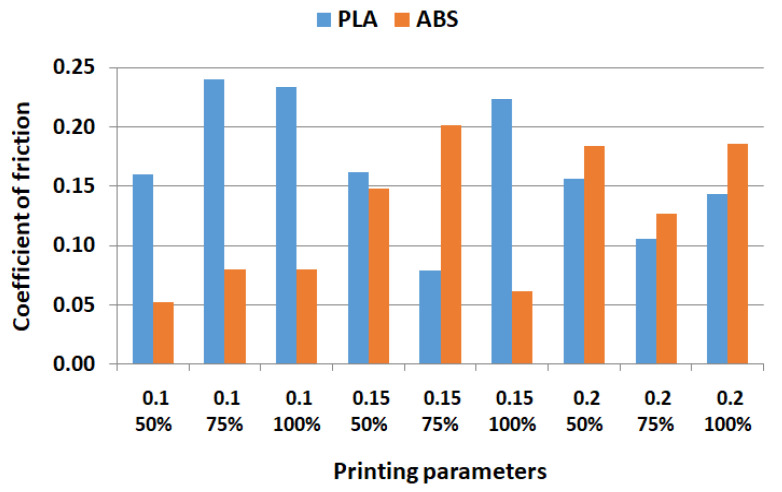
Comparative values for the coefficient of friction.

**Figure 9:**
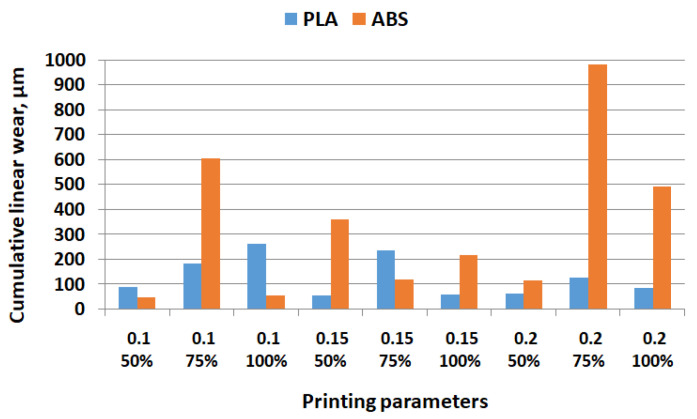
Comparative values for cumulative linear wear.

**Figure 10:**
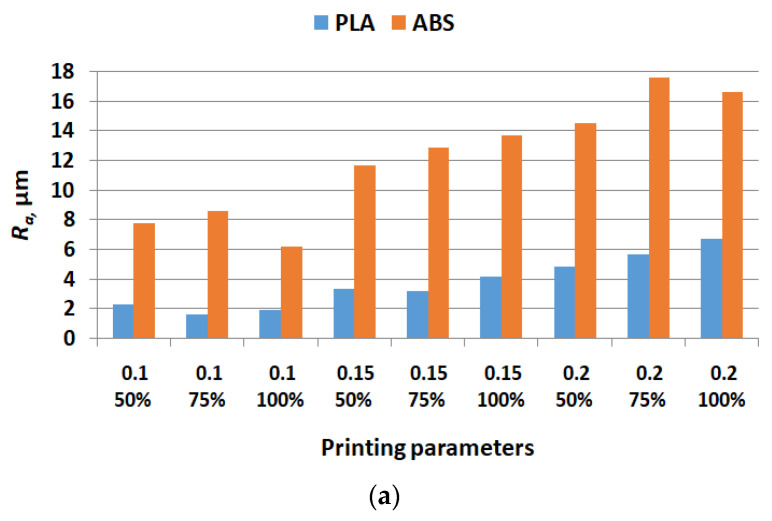

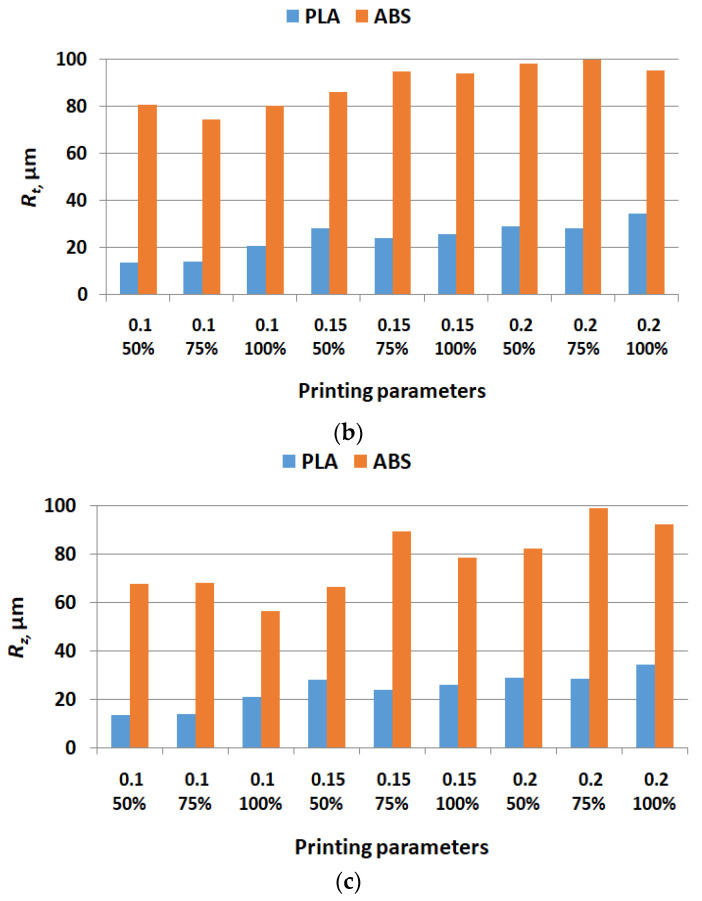
Comparative values for surface roughness parameters:(**a**) *R_a_*—measures within a certain sampling length the average of the peaks and valleys of the metal surface, including the deviation from the mean line; (**b**) *R_t_*—total height of the roughness profile; (**c**) *R_z_*—calculated by measuring the vertical distance from the highest peak to the lowest valley within five sampling lengths, then averaging these distances.

Figure 8, Figure 9 and Figure 10 show the comparative experimental results for the coefficient of friction, cumulative linear wear, and surface roughness parameters.

The comparative results from Figure 8 show, overall, that for smaller layer thicknesses, PLA samples have a greater coefficient of friction compared to ABS. For 0.2 mm layer thickness, the situation is the opposite, a similar conclusion as in [71], where 1 mm layer thickness was used. 

From Figure 9, it can be observed that at a lower layer thickness (0.1 mm), cumulative linear wear is greater for PLA samples, except at 75% infill percentage, where three times greater values for ABS. For 0.2 mm layer thickness, the ABS samples exhibited greater cumulative linear wear: up to seven times greater at 75% infill percentage. Therefore, it is not recommended to use 75% or 100% infill percentage and 0.2 mm layer thickness when printing ABS parts. 

The coefficient of friction and cumulative linear wear for both materials are notably affected by the values of printing parameters. However, a clear and direct relationship of dependence between these parameters and the friction/wear behavior cannot be identified. Consequently, it becomes imperative to conduct an optimization analysis to determine the specific values of printing parameters that result in minimal values for both the coefficient of friction and cumulative linear wear.

The graphics from Figure 10 reveal that all surface roughness parameters have greater values for ABS samples compared with PLA, regardless of the considered printing parameters. Opposite, the results from [71] showed that for longitudinal printing direction, the *R_a_* parameter was greater for PLA samples than for ABS, but an infill percentage of 20% and a layer thickness of 1 mm were used. The parameters *R_t_* and *R_z_* are not strongly influenced by printing parameters, unlike *R_a_* which increases with layer thickness for both analyzed materials. This observation is in accordance with the literature findings [45,46,63], where it is specified that a high layer thickness results in an increased *R_a_*.

**Figure 11:**
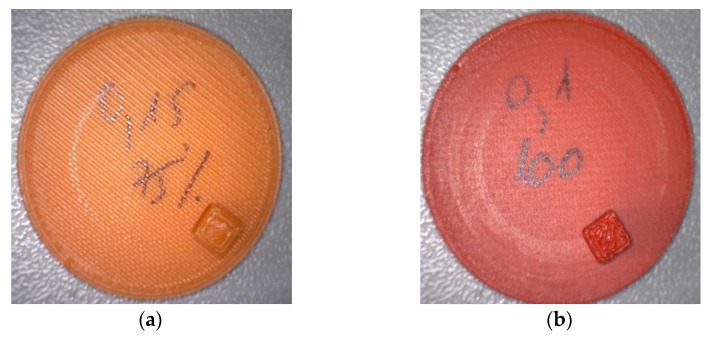
Wear traces during tests: (**a**) ABS samples; (**b**) PLA samples.

Figure 11 presents the wear traces of the samples after experimental testing in order to highlight the patterns and characteristics of wear on the surfaces of these materials.

Uniform wear patterns can be observed for both tested materials and the adhesive–abrasive character of the wear.

**Figure 12:**
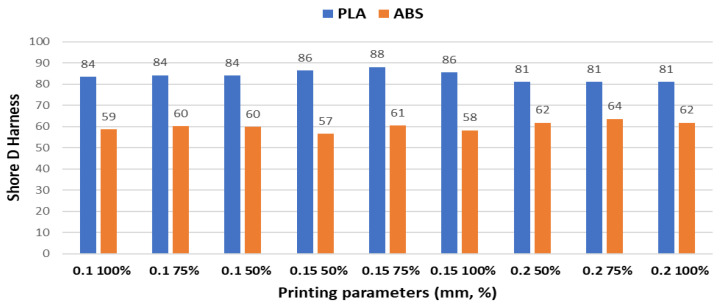
Shore D harness means for PLA and ABS samples.

Figure 12 shows the comparative results regarding the Shore D harness means for PLA and ABS samples. 

Through empirical observation, it is evident that the hardness properties of the polymers PLA and ABS exhibit a consistent trend similar to the friction coefficient and cumulative linear wear. Specifically, the mean hardness of PLA samples is 28.57% higher than that of ABS-printed samples. 

The findings suggest a correlation between the tribological behavior and hardness characteristics of PLA and ABS polymers. Notably, PLA demonstrates a higher resistance to indentation compared to ABS, indicating its superior ability to withstand deformation under external forces.

### 3.2. Optimization of Process Parameters

**Figure 13:**
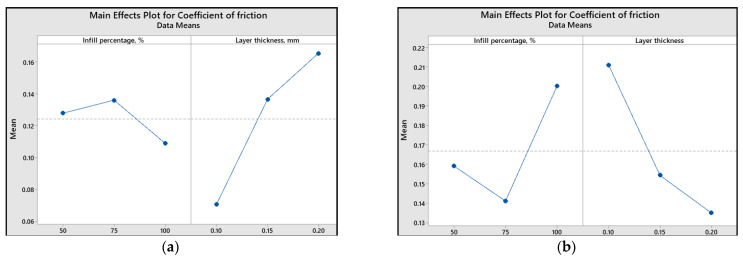
Main effect plots for the coefficient of friction: (**a**) ABS; (**b**) PLA.

**Figure 14:**
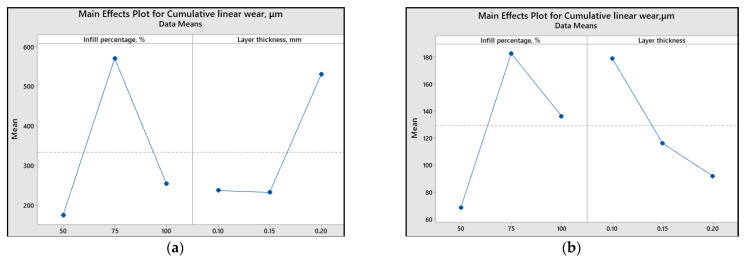
Main effect plots for cumulative linear wear: (**a**) ABS; (**b**) PLA.

**Figure 15:**
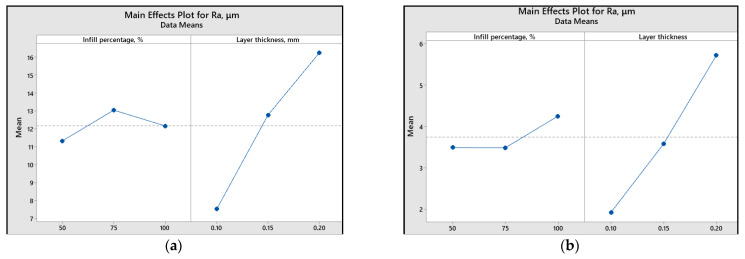
Main effect plots for *R_a_*: (**a**) ABS; (**b**) PLA.

**Figure 16:**
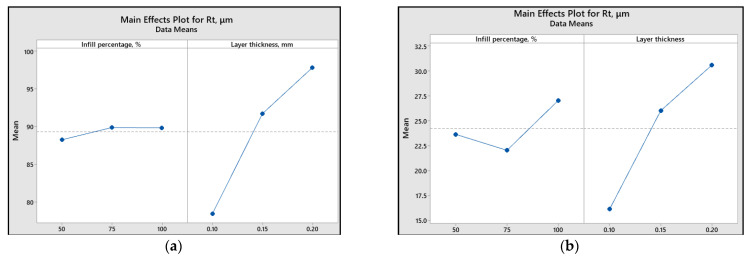
Main effect plots for *R_t_*: (**a**) ABS; (**b**) PLA.

**Figure 17:**
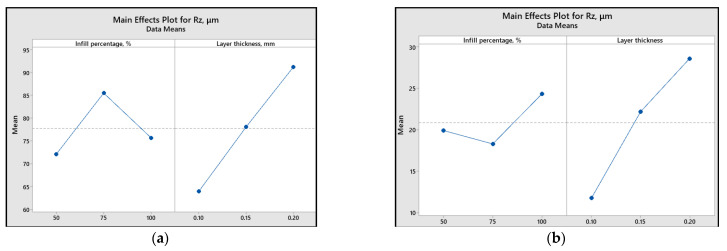
Main effect plots for *R_z_*: (**a**) ABS; (**b**) PLA.

To assess the impact of printing parameters (infill percentage and layer thickness) on different response variables, the main effect plots and graphical representations are illustrated (Figure 13, Figure 14, Figure 15, Figure 16 and Figure 17).

It is observed from the results shown in Figure 13 that the minimum value of the coefficient was obtained for 100% infill percentage and 0.1 mm layer thickness (in the case of ABS 3D-printed samples) and 75% infill percentage and 0.2 mm layer thickness (in the case of PLA 3D-printed samples).

Regarding the minimum value of cumulative linear wear, it can be achieved using 50% infill percentage and 0.15 mm layer thickness (in the case of ABS 3D-printed samples) and 50% infill percentage and 0.2 mm layer thickness (in the case of PLA 3D-printed samples).

Analyzing the plots from Figure 15, Figure 16 and Figure 17, it can be concluded that for both materials, a smaller layer thickness leads to smaller values of surface roughness parameters.

Regarding the influence of infill percentage on the surface roughness, for ABS material, it is recommended to use a value of 50%, while for PLA, 75% infill percentage results in a smoother surface.

Figure 15, Figure 16 and Figure 17 also reveal that the most significant factor influencing the surface roughness is layer thickness. The same conclusion is presented in [46], where it was found that the layer thickness had a contribution percentage for *R_a_* of 51.56%, whereas layer composition contributed only 4.10%.

**Figure 18:**
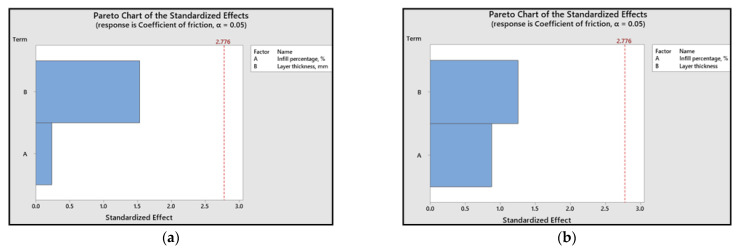
Pareto charts for the coefficient of friction: (**a**) ABS; (**b**) PLA.

**Figure 19:**
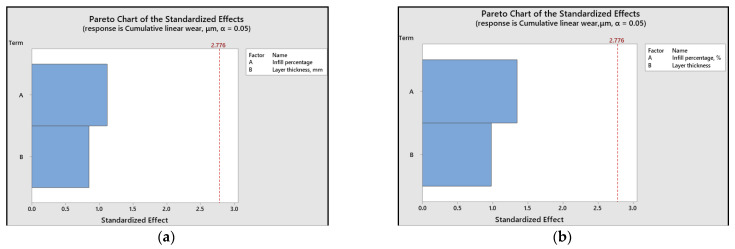
Pareto charts for cumulative linear wear: (**a**) ABS; (**b**) PLA.

The relative significance of printing parameters based on their impact can be effectively illustrated using Pareto charts (Figure 18 and Figure 19). These charts indicate that, concerning the coefficient of friction, layer thickness emerges as the most critical factor, whereas cumulative linear wear is more influenced by the infill percentage.

Furthermore, for ABS 3D-printed samples, the influence of layer thickness on the coefficient of friction value is more pronounced when compared to PLA samples.

**Figure 20:**
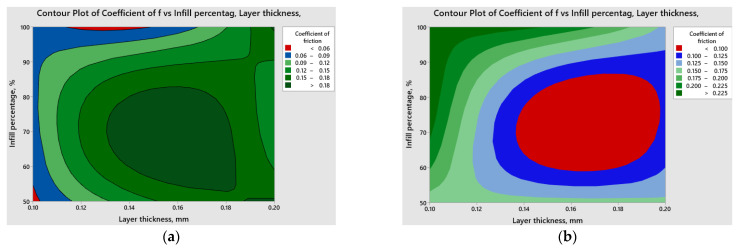
Comparative contour plots for the coefficient of friction: (**a**) ABS; (**b**) PLA.

**Figure 21:**
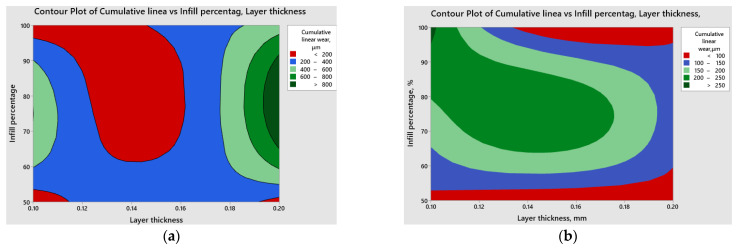
Comparative contour plots for cumulative linear wear (**a**) ABS; (**b**) PLA.

Contour plots derived from Figure 20 and Figure 21 serve as valuable tools for visually grasping the connections between the coefficient of friction and cumulative linear wear with the input parameters. These plots effectively aid in identifying regions characterized by high or low response values, providing a clear and intuitive understanding of the data.

The red contours indicate the regions with lower coefficient of friction and cumulative linear wear, respectively. 

**Figure 22:**
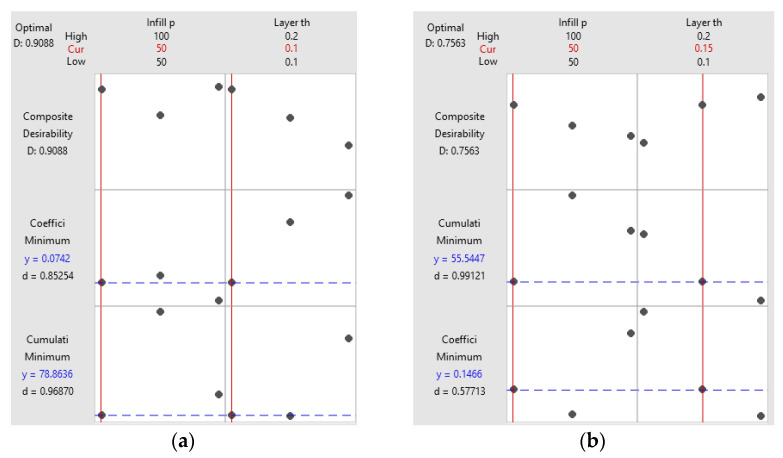
Multi-responseoptimizationplots: (**a**) ABS; (**b**) PLA.

By directing attention to these specific regions, it becomes possible to identify the combinations of variables that lead to more advantageous outcomes concerning the tribological behavior of 3D-printed parts fabricated from ABS or PLA materials, considering certain applications. Based on the above results and considering the variation in both the coefficient of friction and cumulative linear wear in relation to infill percentage and layer thickness, it is recommended to perform a multi-objective optimization to achieve desirable outcomes in terms of coefficient of friction and cumulative linear wear, considered as responses, simultaneously. The optimization criteria are the same for each response, namely to minimize them. Minimizing the coefficient of friction is desirable as it reduces the resistance to motion between surfaces in contact. Lower friction coefficients lead to smoother operation, reduced energy consumption, and less wear and tear on the parts. Additionally, minimizing cumulative linear wear is important for ensuring the durability and reliability of 3D-printed parts. By reducing wear, the parts can maintain their structural integrity and dimensional accuracy over an extended period, resulting in improved performance and operational efficiency. By combining these two objectives into a multi-objective optimization problem, it becomes possible to identify a set of optimal solutions that provide a balance between minimizing the coefficient of friction and cumulative linear wear. This allows the selection of printing parameters that offer the best compromise between these two conflicting objectives, leading to enhanced performance and durability of 3D-printed parts. The multi-response optimization results (illustrated in Figure 22) were obtained using Minitab software.

The optimal combination of printing parameters is 50% infill percentage and 0.1 mm layer thickness (for ABS) and 50% infill percentage and 0.15 mm layer thickness (for PLA).

To validate the results obtained with Minitab software, grey relational analysis optimization methodology for multiple responses optimization was applied.

The Equations (1)–(4) were used, and the corresponding obtained results are presented in Table 3, Table 4, Table 5 and Table 6.

Considering the results analyzed and presented in Table 3, Table 4 and Table 5, Table 6 was obtained to correlate all the information on infill percentage and layer thickness for ABS and PLA. The optimal conditions can be established based on the data from Table 6, corresponding to Rank 1 (the highest value). 

Therefore, it can be seen that the optimal printing parameters for minimum values of coefficient of friction and cumulative linear wear are an infill percentage of 50% and layer thickness of 0.1 mm (for ABS)and an infill percentage of 50% and a layer thickness 0.15 mm (for PLA), as obtained from Minitab response optimization (see Figure 22). 

## 4. Conclusions

The present work aimed to analyze the frictional and wear behavior and surface roughness of 3D-printed PLA and ABS parts. The investigation considered the impact of printing parameters such as infill percentage and layer thickness. The experimentally measured outcomes were the coefficient of friction, cumulative linear wear, and roughness parameters (*R_a_*, *R_z_*, and *R_t_*). Full factorial design of experiments and ANOVA were used to determine the contribution of each input parameter. Multi-objective optimization was carried out to obtain the best values for coefficients of friction and linear wear.

The primary objective of this study was to identify the most effective printing parameters for two specific polymers, PLA and ABS. This was achieved through analysis of the coefficient of friction and cumulative linear wear. The examination of the polymer’s tribological behavior was essential for their application in power transmission, particularly in non-circular gears. The characterization of these polymers in terms of hardness, wear resistance, and coefficient of friction aimed to ensure optimal power transmission and minimize wear in the gear system.

The study revealed a significant impact of 3D printing parameters, specifically infill percentage and layer thickness, on the friction behavior of both ABS and PLA samples. However, the relationship between these parameters proved to be intricate and varied depending on the specific materials being used. Based on the findings from this study, it is not recommended to use a 75% or 100% infill percentage in combination with a layer thickness of 0.2 mm when printing ABS parts. These conditions result in significantly higher linear wear, which could compromise the durability of the 3D-printed ABS components. It is recommended to consider alternative infill percentages or adjust the layer thickness to avoid excessive wear in ABS parts.

The measured data demonstrate that ABS samples consistently exhibit higher surface roughness values (up to three times greater) compared to PLA, regardless of the specific printing parameters being considered. 

Hardness testing was performed to correlate the tribological behavior with hardness values, and the findings strongly indicate a connection between these characteristics both for PLA and ABS polymers. Significantly, PLA exhibits higher resistance to indentation compared with ABS, highlighting its superior capability to withstand deformations when subjected to external forces.

The main effect plots clearly illustrate that the lowest coefficients of friction are achieved under specific printing conditions. For ABS 3D-printed samples, the minimum coefficient of friction was attained with 100% infill percentage and 0.1 mm layer thickness. Conversely, for PLA 3D-printed samples, the minimum coefficient of friction was obtained with 75% infill percentage and 0.2 mm layer thickness. In terms of achieving the minimum linear wear, for ABS 3D-printed samples, 50% infill percentage and 0.15 mm layer thickness should be used. Similarly, for PLA 3D-printed samples, the minimum linear wear can be obtained with a 50% infill percentage and 0.2 mm layer thickness.

The Pareto charts reveal that, for the coefficient of friction, the layer thickness is the most significant factor, whereas the infill percentage has a greater influence on linear wear.

Multi-objective optimization was performed both with Minitab software and using grey relational analysis methodology, and the obtained results were similar. Thus, the optimal printing parameters that yield the minimum values of coefficient of friction and linear wear can be determined as follows: an infill percentage of 50% and layer thickness of 0.1 mm for ABS samples, and an infill percentage of 50% and layer thickness of 0.15 mm for PLA samples.

The application considered in this study was 3D-printed non-circular gears used in flow meters, textile machines, Geneva mechanisms, printing press equipment, pumps, packaging machines, potentiometers, conveyors, windshield wipers, and robotic mechanisms. Power transmission with 3D-printed gears has become a viable option in light industries (textile, food industries, and robotics) due to advancements in 3D printing technology and the availability of sustainable and durable 3D printing materials. The study revealed that the optimal solution was achieved with a 50% infill rather than 100%. This implies that a more efficient material consumption approach can be used in the fabrication of mentioned applications.

Optimizing the printing parameters can help improve the overall performance and durability of 3D-printed parts (in particular non-circular gears), enhancing their tribological behavior, which directly affects their frictional characteristics and wear resistance. However, the optimization function that aimed to minimize both the coefficient of friction and linear wear for 3D-printed ABS and PLA parts encounters several limitations. Firstly, different properties of ABS and PLA materials pose challenges as their responses to infill percentage and layer thickness may vary significantly. Secondly, the complex interaction between these parameters and the targeted results may result in compromises, making it difficult to achieve an optimal balance between friction and wear resistance. Lastly, practical constraints and design considerations could impact the effectiveness of the optimization, necessitating further research and advanced techniques to address these challenges effectively. Overcoming these limitations will contribute to the advancement of additive manufacturing, enhancing the tribological performance and durability of 3D-printed components, so further investigations must be performed.

## Figures and Tables

**Table 1:** The printing parameters described.

Printing Options for 1 Set of Samples	ABS	PLA
Shell width (mm)	1	1
Infill speed (mm/s)	40	70
Estimated print time (min)	60	46
Estimated filament used (g)	10.6	10.6
Extruder temperature (°C)	240	210
Bed temperature (°C)	110	60
Platform addition	Raft only	Raft only

**Table 2:** Parameters and levels used in DOE analysis.

Parameter	Level		
	1	2	3
Infill percentage, %	50	75	100
Layer thickness, mm	0.10	0.15	0.20

**Table 3:** Normalized data responses.

Printing Parameters		Normalized Data			
Infill percentage, %	Layer thickness, mm	ABS		PLA	
		Cumulative linear wear, μm	Coeff. of friction	Cumulative linear wear, μm	Coeff. of friction
50	0.1	1.000	1.000	0.839	0.496
75	0.1	0.404	0.813	0.379	0.000
100	0.1	0.993	0.817	0.000	0.037
50	0.15	0.668	0.361	1.000	0.486
75	0.15	0.924	0.000	0.134	1.000
100	0.15	0.822	0.939	0.978	0.102
50	0.2	0.930	0.115	0.952	0.518
75	0.2	0.000	0.498	0.656	0.833
100	0.2	0.526	0.102	0.850	0.597

**Table 4:** Deviation sequence responses.

Printing Parameters		Deviation Sequence			
Infill percentage, %	Layer thickness, mm	ABS		PLA	
		Cumulative linear wear, μm	Coeff. of friction	Cumulative linear wear, μm	Coeff. of friction
50	0.1	0.000	0.000	0.161	0.504
75	0.1	0.596	0.187	0.621	1.000
100	0.1	0.007	0.183	1.000	0.963
50	0.15	0.332	0.639	0.000	0.514
75	0.15	0.076	1.000	0.866	0.000
100	0.15	0.178	0.061	0.022	0.898
50	0.2	0.070	0.885	0.048	0.482
75	0.2	1.000	0.502	0.344	0.167
100	0.2	0.474	0.898	0.150	0.403

**Table 5:** Grey relational coefficient.

Printing Parameters		Grey Relational Coefficient			
Infill percentage, %	Layer thickness, mm	ABS		PLA	
		Cumulative linear wear, μm	Coeff. of friction	Cumulative linear wear, μm	Coeff. of friction
50	0.1	1.000	1.000	0.756	0.498
75	0.1	0.456	0.728	0.446	0.333
100	0.1	0.987	0.733	0.333	0.342
50	0.15	0.601	0.439	1.000	0.493
75	0.15	0.867	0.333	0.366	1.000
100	0.15	0.737	0.892	0.959	0.358
50	0.2	0.876	0.361	0.912	0.509
75	0.2	0.333	0.499	0.592	0.750
100	0.2	0.514	0.358	0.770	0.553

**Table 6:** Grey relational graded and ranks.

Printing Parameters		I.			
Infill percentage, %	Layer thickness, mm	ABS		PLA	
		Grade	Rank	Grade	Rank
50	0.1	1.000	1 *	0.627	7
75	0.1	0.592	6	0.390	8
100	0.1	0.860	2	0.338	9
50	0.15	0.520	7	0.747	1 *
75	0.15	0.600	5	0.683	3
100	0.15	0.815	3	0.658	6
50	0.2	0.619	4	0.711	2
75	0.2	0.416	9	0.671	4
100	0.2	0.436	8	0.662	5

* the highlighted lines correspond to the optimal values (having Rank 1) of printing parameters for ABS and PLA, respectively.

## Data Availability

Data are contained within the article.
